# CO-INFECTION OF DENGUE AND HEPATITIS A VIRUSES: A RARE CASE REPORT

**DOI:** 10.21010/Ajidv18i2.4

**Published:** 2024-03-08

**Authors:** RODRIGUEZ-SALDAÑA Christian Alberto, FIESTAS-CORDOVA Jessenia, SALDAÑA-FLORES José Gerardo, ABRAMONTE-TENE Walter David

**Affiliations:** 1 Escuela de Medicina, Universidad César Vallejo; 2 Unidad de Neumo-Infecto; Hospital III José Cayetano Heredia, Piura, Perú; 3 Hospital José Cayetano Heredia III-1, Piura; 4 Clinica AUNA Miraflores, Piura

**Keywords:** Co-infection, Dengue virus, Hepatitis A virus, Case report

## Abstract

Dengue fever and hepatitis A are endemic infections caused by viruses that mostly affect developing countries (Volchkova *et al.*, 2016). Co-infection is rare, and represents a diagnostic challenge due to their overlapping symptoms (Yakoob *et al.*, 2009). The febrile syndrome accompanied by abdominal pain and vomiting are the common clinical manifestations of both pathologies. However, confirmation of diagnosis depends on laboratory tests ( Khetarpal and Khanna, 2016; Abutaleb and Kottilil, 2020). We report a case of a young female with dengue and hepatitis A co-infection.

## Case presentation

A 25-year-old female was admitted after 3 days of progressive abdominal pain located in the epigastrium of a stabbing type intensity 9/10, associated with vomiting. Physical examination showed a distended abdomen, increased frequency of bowel sounds, and tenderness in the upper abdominal area on palpation. Dry mucous membranes, skin and sclera with jaundice ++/+++. The following laboratory tests were performed: complete blood count, amylase, lipase, liver profile (albumin, globulins, total and fractionated bilirubin, glutamic oxaloacetic transaminase GOT, glutamic pyruvic transaminase GPT, alkaline phosphatase), urea, creatinine, and urine test. Leukopenia 3500 cells/μl, hemoglobin 11.2 g/dl, hematocrit 33.6%, platelets 175,000 cells/μl, total bilirubin 4.76 mg/dl, direct bilirubin 4.58 mg/dl, albumin 3.4 g/dl, GOT 1989, GPT 2540, leukocytes in urine >100 cells/field and bilirubin in urine 1+ and reactive hepatitis A virus antibodies were found. She was hospitalized and given hydration, antiemetics and analgesics. During her hospital stay, vomiting decreased in frequency, but nausea and fever persisted. On her 5th day of hospitalization, a reactive result was obtained for IgM IgG for dengue positive and NS1 negative.

**Table 1:** Case report’s laboratory test

Test (normal range)	25/12/2022	25/12/2022	26/12/2022	28/12/2022	30/12/2022
	15:19 hrs	20:43 hrs	6:43 hrs	5:58 hrs	8:55hrs
Hemoglobin (12-16)	11.2	11.3	11.3	11.1	12.2
Hematocrit (36-46)	33.6	34.5	34.3	34.4	37.1
Platelets (150000-475000)	175000	171000	180000	209000	290000
Leukocytes (4500-11000)	3500	3530	4010	4740	5350
Neutrophils Seg (1400-6600)	1890	1271	1162	1422	2300
Neutrophils Ab (0-400)	0	0	0	0	0
Eosinophils (0-500)	0	71	80	95	161
Lymphocytes (1300-3500)	1365	1906	2486	2891	2621
Bilirrubin total (0-1.1)	4.76	-	-	5.16	6.25
Bilirrubin direct (0-0.3)	4.58	-	-	4.96	5.92
Bilirrubin indirect (0-0.7)	0.18	-	-	0.2	0.33
Albumine (3.5-5.2)	3.4	-	-	3	3.7
Globuline (2.3-3.5)	3	-	-	2.9	3.9
GPT (0-33)	2540	-	-	1394	1050
GOT (0-32)	1989	-	-	692	541
Alkaline Phosphatase (35-104)	339	-	-	-	720
Serum Urea (17-49)	12	-	-	-	-
Serum Creatinine (0.5-0.9)	0.63	-	-	-	-
C Reactive Protein (0-0.5)	0.27	-	-	0.36	-
Urinary Leukocytes	>100	-	-	-	-
Urinary Reed Blood Cells	03a05	-	-	-	-
Hepatitis B, E Antigen	-	NR	-	-	-
Anti HAV IgM	-	Reactivo	-	-	-
IgM Dengue	-	-	-	-	51.8
IgG Dengue	-	-	-	-	103.1
NS1Ag	-	-	-	-	NR

## Discussion

It is theoretically possible for an individual to be infected with both dengue virus (DENV) and hepatitis A virus (HAV) at the same time, but this is not a common occurrence (Munasinghe and Rajasuriya, 1967). Both viruses are transmitted through different routes and have different symptoms (Shah and Dey, 2015). HAV is primarily transmitted through the fecal-oral route, typically through contaminated food or water. DENV is primarily transmitted through the bite of an infected mosquito, primarily *Aedes* mosquitoes.

In this case report, the patient presented symptoms of both dengue fever and acute liver inflammation, such as fever, headache, muscle and joint pain, rash, and jaundice. If an individual is infected with both viruses simultaneously, the symptoms of each virus may be more severe and have more complications ( Ranathunga *et al.*, 2019; Alemad *et al.*, 2021).

The diagnosis involves laboratory tests, for this case we used serological test against DEV and HAV. For more specific diagnosis, reverse transcriptase polymerase chain reaction (RT-PCR) test is the recommended test to detect the presence of the viruses in the patient’s blood ( Khetarpal and Khanna, 2016; Abutaleb and Kottilil, 2020). However, it has been shown that the IgM antibody exhibits a remarkable specificity, with a positive predictive value greater than 95%, in the acute serum of patients presenting with febrile syndrome in endemic areas, such as in this case from the northern region of Peru (Díaz-Quijano *et al.*, 2006).

Treatment for both viruses is primarily supportive care to manage symptoms, such as pain relief for joint and muscle pain, and fluid replacement to prevent dehydration. Severe cases may require hospitalization and close monitoring. It is important to note that a person infected with either virus should take necessary precautions to prevent spreading of the disease to others, such as washing of hands frequently, practice good hygiene and observe isolation, if necessary.

## Conclusion

It is possible to have a co-infection of DENV and HAV. The outcome of the co-infection would depend on the severity of the symptoms and the overall health of the patient. In some cases, the co-infection may result in more severe symptoms and complications, such as dengue hemorrhagic fever or acute liver failure.

### Conflict of interest

The authors declare that there is no conflict of interest associated with this case report.

### Ethical Consideration

Ethical approval is not required due to the fact that this is a case report.

**List of abbreviations:**
DENV: Dengue virus
HAV: Hepatitis A virus
GOT: glutamic oxaloacetic transaminase
GPT: glutamic pyruvic transaminase
IgM: Immunoglobulin M
IgG: Immunoglobulin M
NS1: nonstructural protein 1
RT-PCR: reverse transcriptase polymerase chain reaction.
